# The emerging roles of MAPK-AMPK in ferroptosis regulatory network

**DOI:** 10.1186/s12964-023-01170-9

**Published:** 2023-08-14

**Authors:** Xinyue Wang, Xiao Tan, Jinping Zhang, Jiaping Wu, Hongjuan Shi

**Affiliations:** 1 Hubei Key Laboratory of Tumor Microenvironment and Immunotherapy, China Three Gorges University, Yichang, 443002 China; 2 College of Basic Medical Science, China Three Gorges University, Yichang, 443002 China

**Keywords:** MAPK, AMPK, Ferroptosis, Autophagy, p53

## Abstract

Ferroptosis, a newform of programmed cell death, driven by peroxidative damages of polyunsaturated-fatty-acid-containing phospholipids in cellular membranes and is extremely dependent on iron ions, which is differs characteristics from traditional cell death has attracted greater attention. Based on the curiosity of this new form of regulated cell death, there has a tremendous progress in the field of mechanistic understanding of ferroptosis recent years. Ferroptosis is closely associated with the development of many diseases and involved in many diseases related signaling pathways. Not only a variety of oncoproteins and tumor suppressors can regulate ferroptosis, but multiple oncogenic signaling pathways can also have a regulatory effect on ferroptosis. Ferroptosis results in the accumulation of large amounts of lipid peroxides thus involving the onset of oxidative stress and energy stress responses. The MAPK pathway plays a critical role in oxidative stress and AMPK acts as a sensor of cellular energy and is involved in the regulation of the energy stress response. Moreover, activation of AMPK can induce the occurrence of autophagy-dependent ferroptosis and p53-activated ferroptosis. In recent years, there have been new advances in the study of molecular mechanisms related to the regulation of ferroptosis by both pathways. In this review, we will summarize the molecular mechanisms by which the MAPK-AMPK signaling pathway regulates ferroptosis. Meanwhile, we sorted out the mysterious relationship between MAPK and AMPK, described the crosstalk among ferroptosis and MAPK-AMPK signaling pathways, and summarized the relevant ferroptosis inducers targeting this regulatory network. This will provide a new field for future research on ferroptosis mechanisms and provide a new vision for cancer treatment strategies.

Video Abstract

**Supplementary Information:**

The online version contains supplementary material available at 10.1186/s12964-023-01170-9.

Over the past few decades, therapies that induce cancer cells to initiate a regulatory cell death program have attracted widespread attention. Regulatory cell death [1] is a proactive process and can occur through a range of molecular mechanisms and signaling pathways that mediate it. Since the discovery of ferroptosis in 2012 [2], the study of the mechanisms of regulatory cell death modalities has once again gained new hotness. Ferroptosis is a new form of regulated cell death, which along with elevated iron ion levels and driven by peroxidative damages of polyunsaturated-fatty-acid-containing phospholipids in cellular membranes [2–4]. Although ferroptosis is morphologically, biochemically and genetically different from other death programs such as apoptosis, necrosis, and autophagy. Yet, it was recently found that the occurrence of ferroptosis is accompanied by autophagy-like changes so that is considered to be an autophagy-dependent cell death in a variety of cells [5]. The fact that autophagy-related factors (e.g., BECN1, mTOR, AMPK) can regulate specific regulatory ferroptosis networks also provides us with a new idea as to whether the occurrence of ferroptosis is somehow related to the signaling pathways that regulate autophagy.

Ferroptosis occurs with the development of oxidative stress and energy stress [6], and the activity of cancer-related signaling pathways can regulate these two stress responses. The dysregulation of the mitogen-activated protein kinase (MAPK) and AMP-activated protein kinase (AMPK) pathways, which represent the regulation of oxidative stress and energy stress, is critical for ferroptosis. Mitogen-activated protein kinases (MAPKs) family which include extracellular signal-regulated kinase (ERK), c-Jun N-terminal kinase (JNK) and p38mitogen-activated protein kinase (p38 MAPK), are a group of signaling molecules that play a critical role in the regulation of cell growth, differentiation, apoptosis and oxidative stress response. The study found that the high ROS environment will mediate MAPK signaling pathway, inducing ferroptosis [7]. Aberrant activation of the MAPK pathway is prominently implicated in cancer development and progression. The occurrence of ferroptosis is accompanied by the accumulation of lipid peroxides, which can induce energy stress that depletes ATP to cause cell death. AMPK is a sensor of cellular energy status, affects cellular redox homeostasis and iron metabolism, and related studies have found that promoting AMPK activation through energy stress can inhibit ferroptosis [6]. Under physiological conditions, AMPK plays a major role in biological processes such as cell growth, autophagy, and apoptosis. While it is interesting that the effects of AMPK activation on ferroptosis involve modulation of autophagy, mTORC1 signaling, p53 activation, cystine uptake, or iron metabolism which can induce ferroptosis. Moreover, recent studies have demonstrated that inhibition of MAPK pathway activity can activate the LKB1-AMPK signaling pathway [8]. These intricate networks make MAPK and AMPK pathways to be enigmatic in regulating the onset of ferroptosis.

## An overview of ferroptosis

Dolma's et al. [9] discovered a new compound erastin which can induce cancer cell death differently from apoptosis in 2003. Furthermore, in 2008, Stockwell's et al. found that this unique mode of cell death appeared when treating cancer cells with two compounds, erastin and RSLs, in a high-throughput screening study of oncogenes molecules [10]. Dixon et al. named this unique new modality of regulated cell death (RCD) ferroptosis in 2012 [2], which is characterized by the accumulation of lipid reactive oxygen species (ROS) and iron-dependent. The morphological change is manifested with mitochondrial atrophy, cristae disappearance or reduction, increased membrane density, and outer membrane rupture. Compared to normal cells, cells in ferroptosis are much smaller, which differs from apoptosis, necrosis and autophagy [2]. However, recent studies have shown that under certain conditions, other forms of cell death can occur along with ferroptosis. In addition, various studies have confirmed the critical role of the MAPK signaling pathway in regulating ferroptosis. The active factors of ferroptosis can affect glutathione peroxidase through different pathways, leading to decreased intracellular antioxidant capacity and accumulation of lipid ROS [11].

## An overview of MAPK

**Fig. 1:**
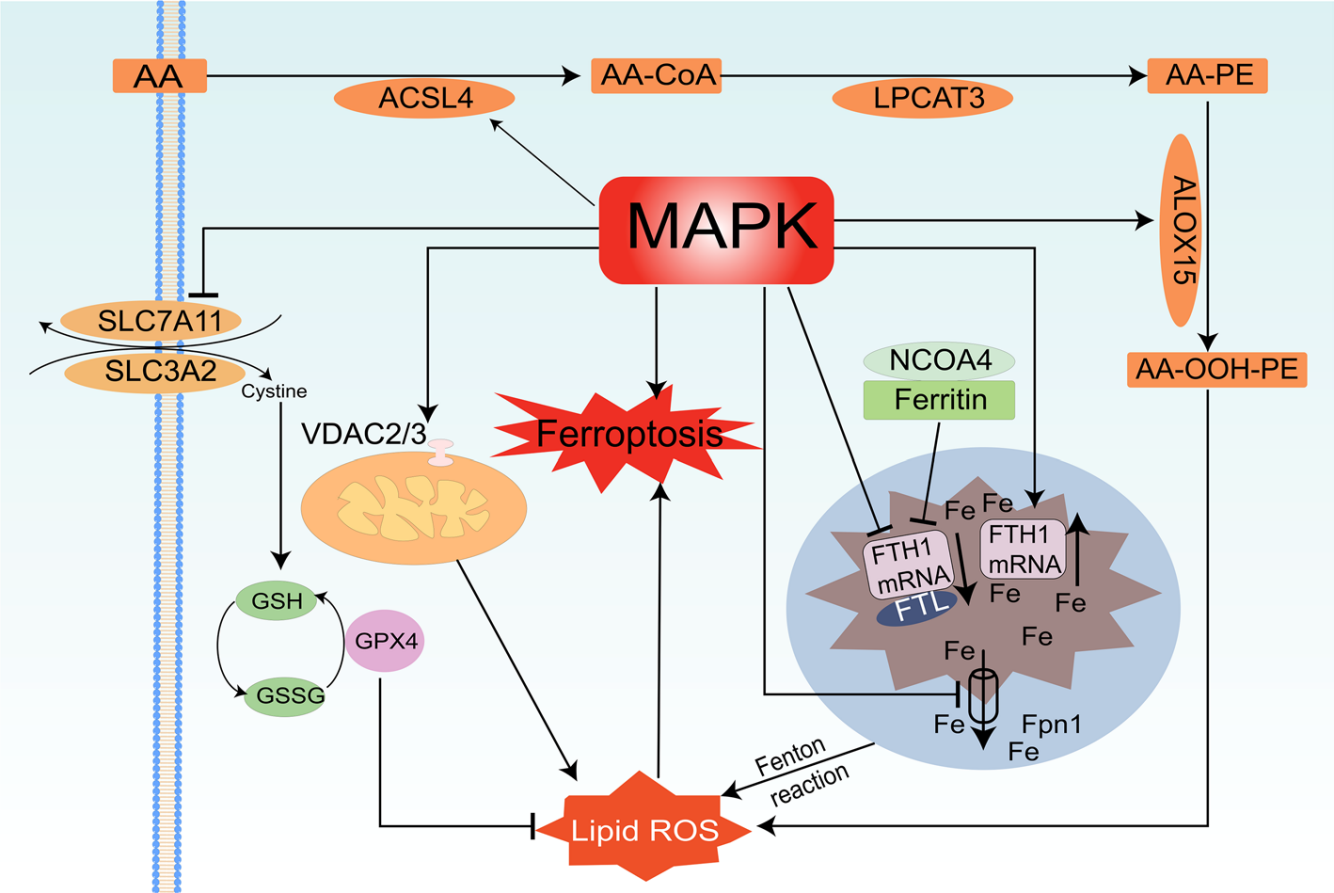
Molecular mechanism of MAPK regulation of ferroptosis

The exact mechanism of MAPK pathway regulating ferroptosis has not been elucidated. Here, we mainly describe the molecular mechanism of MAPK signaling pathway involved in iron ion, lipid and amino acid metabolism to regulate ferroptosis. (Fig. 1).

As one of the highly conserved signaling pathways involved in various biological functions, the MAPK cascade plays an important part in the signaling module of eukaryotes [12]. The MAPK kinase kinase (MAPKKK or MEKK), a MAPK kinase (MAPKK or MEK), and a MAPK (MPK) have constituted a complete MAPK cascade. It is known that MAPK kinases can be activated by tandem phosphorylation of three or more protein kinases to regulate the activity of transcription factors and the expression of corresponding genes, thereby causing cellular responses and participating in the regulation of cell proliferation, differentiation, transformation and apoptosis, and that the activation of MAPKs is closely associated with various diseases such as inflammation and tumors [12]. Four MAPK signaling pathways have been identified in mammalian cells, including the ERK 1/2 signaling pathway, the JNK signaling pathway, the p38 signaling pathway and the ERK5 significant mitogen-activated protein kinase pathway [13].

Related studies have confirmed that the MAPK signaling pathway plays an important role in the sensitivity of some cancer cells to ferroptosis [2]. RAS-RAF-MEK-ERK is the most classical signaling pathway of MAPK. MEK activity is one of the determinants of ferroptosis, and the activation of ERK1/2 is the characteristic of ferroptosis. As an upstream protein of ERK1/2 activation [2], there is a complex relationship between RAS activity and ferroptosis. When examining the correlation between RAS mutation status and the potential of erastin-induced ferroptosis, erastin can induce ferroptosis in RAS-activated cancer cell lines, not RAS wild-type group. Compared with BJ-TERT/LT/ST cells lacking the oncogene RAS gene, erastin induced stronger ferroptosis in HRAS-mutated BJ-TERT/LT/ST/RAS^V12^cells [14]. Furthermore, it was confirmed that HL-60/NRAS Q61L (NRAS high expression) cells are more sensitive to erastin-induced ferroptosis than HL-60 (RAS wild-type) cells [15]. Moreover, sensitivity to erastin-induced ferroptosis was also reduced in shRNA-mediated KRAS-silenced Calu-1 lung cancer cells, and inhibition of BRAF activity was also found to reduce sensitivity to erastin in BRAF-silenced A-673 cells [16]. In addition, diffuse large B-cell lymphoma (DLBCL) and renal cell carcinoma cell lines (RCC) without mutations in the RAS signaling pathway showed a higher sensitivity to ferroptosis [14]. These findings have confirmed that the activity of MAPK signaling pathway can regulate ferroptosis.

### MAPK regulates ferroptosis through iron ion metabolism

The metabolism of iron ions is closely related to ferroptosis, the massive accumulation of free iron ions is also the causative factor and manifestation of the occurrence of ferroptosis [2, 17]. By controlling both the input of iron ions and the storage capacity of ferritin can affect the iron ion in a metabolic pathway, which ultimately ripples through the occurrence of ferroptosis [18].

The transferrin receptor 1 (TfR1) acts as a "transport vehicle" for iron ions, in which the TF-Fe^3+^ complex "rides" and binds to TfR1, leading to ferroptosis by transferring extracellular iron into the cell and increasing intracellular iron ion content [19, 20]. As an essential iron regulatory protein, TfR1 overexpression induces ROS generation through the Fenton reaction and ultimately promotes ferroptosis [21, 22]. A related study concluded that RAS activation can enrich the intracellular iron pool through the upregulation of TfR1 [10]. Furthermore, it was found that a mitogen response element in the 5' untranslated region (UTR) of TfR1 mRNA, indicating that RAS-RAF-MEK-MAPK signaling pathway may upregulate the expression of TfR1 [23, 24]. In addition, in the fibroblast-derived cell lines BJ-TERT, BJ-TERT/LT/ST, and BJ-TERT/LT /ST/RAS ^V12^ (HRAS activated), it was found that the mRNA expression of TfR1 increased gradually in these three cell lines. In contrast, knockdown of TfR1 in BJ-TERT/LT/ST/RAS ^V12^ cells partially inhibited ferroptosis induced by erastin [10] Related studies have confirmed that the JNK/p38MAPK pathway plays a role in erastin-induced ferroptosis in HL-60/NRAS Q61L cells and that activation of JNK/p38MAPK, an upstream signaling pathway of TfR1, can upregulate the expression level of TfR1 [25]. Moreover, during ascorbic acid (AA)-induced apoptosis in human gastric cancer cells AGS, it was found that inhibition of p38-MAPK could inhibit the upregulation of TfR1, thus reducing intracellular iron ion storage. This also reinforces the important role of MAPK signaling pathway activity on TfR1 regulation. In conclusion, the activation of the MAPK signaling pathway stimulates expression of TfR1, leading to increased intracellular iron ions, ultimately leading to ferroptosis.

In addition to the TfR1-mediated iron uptake pathway, intracellular iron homeostasis can be regulated by ferritin phagocytosis [26]. Ferritin consists of ferritin heavy chain 1 (FTH1) and ferritin light chain (FTL), which are in charge of the storage of free intracellular iron ions [27]. FTH1 can convert Fe ^2+^ into Fe ^3+^, binds to FTL to reduce the toxicity of intracellular Fe ^2+^, thus preventing ferroptosis to occur. Therefore, inhibiting the expression of FTH1 can effectively induce the occurrence of ferroptosis [28]. Related studies have confirmed that overexpression of nuclear receptor coactivator (NCOA4) suppresses the expression level of FTH1, thereby enhancing the autophagic degradation of ferritin [26, 29], which provided a substrate for ferroptosis [30, 31]. Related studies have confirmed that over expression of NCOA4 in PANC1 cells inhibited FTH1 expression, thereby inducing ferritin degradation and ferroptosis [26]. Therefore, inhibition of FTH1 expression levels can induce ferroptosis [32]. Meanwhile, reduced levels of FTH1 expression were found in the RAS-mutated BJ-TERT/LT/ST/RAS V12 cell line, indicating that mutations in RAS can suppress the expression of FTH1 [10]. In the mechanism of Cadmium telluride quantum dots (CdTe QDs) induced inflammation in macrophages, it was found that the action of CdTe QDs promoted the phosphorylation of ERK1/2 and the degradation of FTH1 in lysosomes, which in turn activated the autophagy of ferritin [33]. In summary, activation of the MAPK pathway induces ferroptosis by enhancing the degradation of FTH1 and thus the autophagic process of ferritin.

Ferroportin1 (Fpn1) is the unique iron export protein that regulates intracellular iron [34]. Deficiency of Fpn1 leads to the accumulation of iron in cells, and excess iron causes Fenton reaction, which increases lipid ROS accumulation, thereby inducing ferroptosis. Disturbances in iron metabolism are typical of subarachnoid hemorrhage (ASH), and in the treatment of ASH with oncostatin M (OSM, an iron-regulator inducer) it was found that the expression of hepcidin was increased and the expression level of Fpn1 was decreased by the action of OSM, enhancing the sensitivity to ferroptosis [35]. Meanwhile, the study has found that the knockdown of Fpn1 could enhance erastin-induced ferroptosis in neuroblastoma [36]. Related studies have verified that activation of the ERK/MAPK pathway can induce Fpn1 expression [37]. Meanwhile, it was found that using a MAPK inhibitor (U0126) can inhibit Fpn1 expression in a study of fasting [38]. Therefore, inhibition of the MAPK signaling pathway can reduce the expression level of Fpn1, which mediates impaired iron export and thus induces the development of ferroptosis.

### MAPK regulates ferroptosis through lipid metabolism

Another characteristic of ferroptosis is the accumulation of lipid peroxides [2]. On the one hand, ferroptosis is induced by the Fe^2 +^ dependent Fenton reaction, which reacts with hydrogen peroxide to generate highly reactive hydroxyl radicals by oxidizing Fe^2+^ to Fe^3+^. On the other hand, the oxidation of free PUFA, phosphatidylethanolamine (PE), cardiolipin, and phosphatidylcholine through the enzymatic reactions leads to the accumulation of lethal reactive oxygen species such as superoxide, hydrogen peroxide, hydroxyl radicals and lipid peroxides which leads to the occurrence of ferroptosis [4, 39]. Free PUFA is present in the cell membrane, lysosome membranes and the endoparasitic reticulum that containing arachidonic acid (AA), epinephrine acid and ω-6 fatty acids which containing PE. Acyl-CoA synthetase long-chain family member 4 (ACLS4) and lysophosphatidylcholine acyltransferase 3 (LPCAT3) are responsible for the integration of PUFA into the cell membrane [40], which are two key players in the regulation of PUFA synthesis and remodeling [41]. In the process of ferroptosis, AA can be esterified to AA-PE under the action of ACSL4 and LPCAT3, which is then oxidized to lipid peroxides by arachidonate lipoxygenase 15 (ALOX15) [41]. ALOX15- induced phospholipid oxidationacts as a “burning point” to ignite ferroptosis signals.

ADP-ribosylation factor 6 (ARF6) is a fellow of the RAS superfamily, which encodes a small guanine nucleotide-binding protein (GTP-binding protein) involved in the regulation of cancer cell invasion, metastasis and proliferation [42]. A related study found that knockdown of ARF6 in pancreatic cancer cell lines strongly enhanced the sensitivity of pancreatic cancer PANC-1 cell lines to RSL3-induced ferroptosis and was accompanied by an increase in the protein expression level of ACSL4 [43]. Meanwhile, a negative correlation between ARF6 and ACSL4 expression was also confirmed in tissue specimens from pancreatic cancer patients. Interestingly, although ARF6 silencing upregulated the protein level of ACSL4, it did not inhibit the mRNA level of ACSL4, suggesting that ARF6 regulates ACSL4 by acting at the post-translational level [43]. Thus, the RAS/MAPK pathway can induce ferroptosis by enhancing the expression of ACSL4, which in turn affects the PUFA oxidation process and ultimately leads to the accumulation of lipid peroxides. In addition, the small scaffold protein Raf1 kinase inhibitory protein (RKIP1) as the inhibitory protein of the Raf1 kinase cascade reaction could bind to ALOX15 [44], and enhance the oxidation of AA-PE by ALOX15, thereby promoting lipid peroxidation and ferroptosis [45]. Taken together, inhibition of MAPK pathway can induce ferroptosis by regulating factors related to lipid metabolism.

### MAPK regulates ferroptosis through amino acid metabolism

Another characteristic manifestation of ferroptosis is the impaired synthesis of the antioxidant and free radical scavenger glutathione (GSH) in vivo [46]. The cystine/glutamate transporter (system Xc-) maintains glutathione homeostasis by regulating glutathione and by transporting glutamate, it is known that inhibition of its activity can lead to imbalance in amino acid metabolism, preventing the normal entry of extracellular cystine into the cell, thus affecting GSH synthesis, causing inhibition of GPX4 activity, reduction of cellular antioxidant capacity and accumulation of lipid ROS thereby inducing ferroptosis [47]. System Xc ^−^ is a disulfide-bonded heterodimer composed of a non-glycosylated light chain xCT (SLC7A11) and a glycosylated heavy chain CD98hc (SLC3A2) [48]. We can know that transcriptional regulation of light chain SLC7A11 is essential for systemic Xc- activity [49, 50], while heavy chain SLC3A2 is responsible for anchoring SLC7A11 to the cell membrane and maintaining the stability of SLC7A11 protein [51]. It was found that the inactivation of SLC7A11 can induce the occurrence of ferroptosis. In contrast, overexpression of SLC7A11 increases the activity of system Xc^−^ and promotes GSH biosynthesis, resulting in ferroptosis resistance in cancer cells [2, 52, 53].

It was noted that the RAS-RAF-MEK-ERK signaling pathway, a classical signaling pathway of MAPK, can regulate the transcription of SLC7A11. In the chimeric tyrosine kinase (3T3 EN) transformed 3T3 KRAS ^V12^ cell line [54, 55], and under exogenous oxidative stress conditions when tracking SLC7A11 expression using the system Xc- tracer (18)F-5-fluoroaminosuberic acid ((18)F-FASu) [56, 57], it was found that the expression and activity of SLC7A11 appeared upregulated in the 3T3 KRAS V12 cell line compared to the normal cell line [58]. Meanwhile, ERK activity was inhibited using MAPK pathway inhibitors, SLC7A11 expression levels were found to be reduced [58]. Taken together, activation of MAPK pathway can upregulate the expression level of SLC7A11. More interestingly, gene enrichment analysis (GSEA) revealed that the regulation of SLC7A11 by RAS-RAF-MER-ERK acts through an ERK substrate, Ets-1 [58]. And the immunoprecipitation (ChIP) results also validated this prediction. Correlated studies confirmed that under exogenous oxidative stress conditions, endogenous Ets-1 regulates the expression level of SLC7A11 by binding through the promoter region of SLC7A11 [58]. In addition, luciferase trans-activation assays showed that exogenous Ets -1 activated the luciferase reporter gene containing the SLC7A11 promoter in a concentration-dependent manner. Thus, Inhibition of MAPK signaling pathway may inhibit SLC7A11 expression through the action of Est1, impairs the transport activity of systemic Xc^−^, and thus induces ferroptosis.

### Other

The MAPK pathway can affect the occurrence of ferroptosis by regulating the metabolism of iron ions, lipids and amino acids, but it can also regulate ferroptosis through other factors.

The nuclear factor erythrocyte-related factor 2 (Nrf2) is the critical transcription factors of the antioxidant response, prevents lipid peroxidation and ferroptosis by increasing the transcription of a series of antioxidant-responsive genes [59], such as NAD(P)H, quinone oxidoreductase 1 (NQO1), heme oxygenase-1 (HO-1), glutathioneS-transferaseA1(GSTA1), glutamate-cysteineligase(γGCS) [60]. Related studies have demonstrated that activation of Nrf2/HO-1 can effectively mitigate ferroptosis, thus inhibition of the Nrf2/HO-1 activity can be a potential target in ferroptosis [61, 62]. Keap1 is one of the major upstream regulators of Nrf2 which is mainly responsible for regulating the subcellular localization and homeostasis of Nrf2 [63]. It was found that overexpression of Keap1 can repress the nuclear accumulation and transcriptional activity of Nrf2 [64]. The tight binding of Keap1 to Nrf2 prevents the activation of Nrf2, while in the presence of external stimuli or oxidative stress, p62 protein accumulates on the mitochondrial membrane and binds to Keap1, p62-mediated Keap1 degradation to promote phosphorylation of Nrf2, translocate it into the nucleus, and then bind to anti-oxidative stress genetic elements to resistance ferroptosis [65]. Taken together, inhibition of p62-Keap1-Nrf2 system may represent an attractive approach for induce ferroptosis.

Nrf2 activation has turned out to be regulated by the mitogen-activated kinases (MAPK), including ERK, JNK and p38 MAPK [66, 67]. Related studies have demonstrated that Nrf2 is a novel substrate for pERK and pERK can directly phosphorylate Nrf2, promoting activity and nuclear accumulation of Nrf2 [68]. Realgar is a traditional Chinese medicine (TCM), which can induce neuronal cell damage through activation of ERK and p38 signaling pathways. In this mechanistic study, it was found that inhibition of the activity of p38 and ERK signaling pathways resulted in enhanced binding of p62 to Keap1, which inhibited Nrf2 activity to some extent [69]. In the study of the mechanism of action of β-Phenethyl Isothiocyanate (PEITC) induces cell death in human osteosarcoma through, activation of MAPK signaling pathways, PEITC decreased p62 expression which promoting nuclear accumulation of Nrf2 [70]. The physically-made gold nanoparticle (GNP) can induce cytosolic Nrf2 translocation into the nucleus, while the GNP-induced Nrf2 activation will reduce by JNK and p38 MAPK inhibitors [66]. Nrf2 is phosphorylated in vivo at multiple sites by MAPKs, while it is interesting that the MAPKs does not affect the interaction between Nrf2 and Keap1 in vivo. However, MAPK regulation of Nrf2 is not achieved through direct phosphorylation of Nrf2, and the possible pathway is through translational regulation of protein synthesis of Nrf2 [71]. In summary, activation of the MAPK pathway can inhibit ferroptosis by promoting the phosphorylation and nuclear translocation of Nrf2.

The MAPK signaling pathway can also regulate ferroptosis by regulating voltage-dependent anion channels (VDAC) between the mitochondria and the cytoplasm [3]. VDAC plays an important role in mitochondrial dysfunction and accumulation of lipid peroxides [2, 16, 72]. In the quiet state, VDACs are blocked by tubulin, and erastin can reverse the blocking of VDACs by liberating tubulin, thereby opening VDACs and leading to a considerable accumulation of ROS. Furthermore, cells in which the RAS/MAPK signaling pathway is activated, the opening level of VDACs increases, thereby enhancing the sensitivity of ferroptosis induced by erastin [16, 73].

## Inhibition of MAPK induces ferroptosis through an intermediate bridge AMPK

As a widespread energy sensor in eukaryotes, AMPK is a heterotrimeric complex composed of a catalytic α subunit and two regulatory subunits, β and γ which is responsible for regulating cellular metabolism. The intracellular ADP/ATP ratio regulates the activation of AMPK, ADP or AMP binds to the γ subunit and phosphorylates the threonine (Thr) 172 residue on the α catalytic subunit through upstream protein kinases [74]. Activation of AMPK can phosphorylates downstream signaling factors and inactivate the ATP anabolic pathway; conversely, inhibition of AMPK will activate the catabolism of ATP [75]. Related studies confirm that activation of AMPK enhances GPX4-dependent ferroptosis [76].

Live kinase B1 (LKB1) and Ca (2 +)/calmodulin-dependent protein kinase kinase 2 (CAMKK2) are two crucial upstream kinases for activating AMPK by phosphorylating the Thr172 residue of AMPK [77, 78]. LKB1 positively regulates AMP-activated protein kinase. After forming a complex with Strad and MO25, LKB1 is translocated from the nucleus to the cytoplasm, regulates the activity of AMPK-related downstream kinases, which participates in cell growth metabolism and energy regulation. Moreover, MO25α plays a key role in stabilizing the LKB1 activation loop in a conformation required for phosphorylation of substrates [79]. Concerning regulation of AMPK by LKB1-mediated phosphorylation [80],when energy utilization changes, in other words the ratio of ATP /ADP or ADP/AMP changes, excess AMP binds to the γ -subunit of AMPK, and LKB1 activates AMPK kinase activity by phosphorylating Thr-172 in the α-subunit of AMPK [81]. Related studies have demonstrated that the LKB1-AMPK pathway has an important role in promoting cellular perception of bioenergetic contingencies and adapting to promote tumor cell survival. LKB1 acts as a critical factor to activate AMPK, while there is ample evidence that inhibiting the MAPK can activate the LKB1-AMPK signaling pathway. The study indicated that ERK and AMPK activities were found to be negatively correlated in melanoma cells harboring the B-RAF V600E. The phosphorylation of LKB1 by ERK and ribosomal protein S6 kinase (RSK) which are the kinases downstream of B-RAF impairs the ability of LKB1 to associate with and phosphorylate AMPK at Thr172, so that the activation of AMPK is blocked. Meanwhile, AMPK activity increases when using MEK inhibitors on the B-RAF-activated melanoma cells [82]. Some experiments have shown that following the use of MEK inhibitors in immortalized mouse embryonic fibroblasts (MEFs) of LKB1^+/+^ and LKB1^−/−^, it was found that MEFs of LKB1^+/+^ lead to activation of AMPK, MEFs of LKB1^−/−^ did not show this phenomenon [82]. This indicates that inhibition of the MAPK pathway activation of AMPK is mediated through LKB1. In the study on the pathogenesis of nasopharyngeal carcinoma (NPC), it was found that Epstein-Barr virus (EBV) plays a vital role as the prominent oncogene encoded by EBV, latent membrane protein 1 (LMP1),which inhibits LKB1-AMPK activity through ERK-MAPK activated by CTAR1 domain of LMP1 [83]. HRAS hyperactivation inhibits the LKB1/AMPK pathway, the mutant HRas p.G12A and p.G12S stimulates HRas activation and inhibits the expression of the AMPKα2, which in turn leads to inhibition of AMPK activity, this process is accompanied by a decrease in LKB1 protein levels [84]. In summary, inhibiting the activity of the MAPK pathway can activate the LKB1-AMPK signaling pathway, thereby induce ferroptosis.

### AMPK induces ferroptosis through autophagy

Although ferroptosis has been defined as a new regulatory cell death mode different from apoptosis and autophagy, recent studies have shown that activation of autophagy can induce ferroptosis in normal and cancer cells [32, 85]. Autophagy can promote ferroptosis by selectively degrading anti-ferroptosis regulators and ferritin in cancer cells [32]. Excessive autophagy can promote ferroptosis by increasing iron ion content and lipid peroxide accumulation [86]. Autophagy can also induce ferroptosis by regulating iron metabolism or lipid metabolism through degradation of GPX4, lipid droplets and ARNTL/BMAL1 (aryl hydrocarbon receptor nuclear translocator-like).

Inhibition of the MAPK signaling pathway can activate the LKB1-AMPK signaling pathway, then activate autophagy. A related study revealed that activated AMPK maintains cellular amino acid homeostasis by controlling the mechanistic target of the central sensor of cellular amino acids, rapamycin complex 1 (mTORC1) [87]. While the mTORC1 is composed of three core components: mTOR, mLST8 (mammalian lethal with SEC13 protein 8) and RAPTOR (a mTOR-associated regulatory protein) [88]. A related study found that Sirtuin 3 (SIRT3), a typical NAD^+^-dependent mitochondrial protein deacetylase, plays an important role in ROS generation and cell death [89]. SIRT3 can directly inhibit the activity of GPX4 to induce the onset of ferroptosis. Meanwhile, when mitochondrial dysfunction occurs, the increase in SIRT3 expression level promotes autophagy through activation of AMPK-mTOR pathway, which ultimately induces ferroptosis in trophoblast cells [90]. Meanwhile, it was found that the benzopyran derivative 2-imino-6-methoxy-2H-chromene-3-carbothioamide (IMCA) induced ferroptosis in colorectal cancer (CRC) by activating the AMPK signaling pathway and inhibiting the mTOR/p70S6k signaling pathway activity, downregulation of SLC7A11 expression and reduce of Cys and glutathione, ultimately leading to ROS accumulation and ferroptosis [91]. Meanwhile, we found that ERK activation inhibited LKB1/AMPK activity and thus increased mTOR activity by phosphorylating LKB1, which also demonstrated that LKB1/AMPK activity was negatively correlated with mTOR, in our study of the mechanism of ERK-dependent myofibrillar transformation caused by IL11 [92]. Other related studies also confirmed the negative relationship between AMPK and mTOR [93]. It was found that when cellular energy level is low, the LKB1 phosphorylates and activates AMPK to inhibit mTORC1. The inhibitory effect of AMPK on mTORC1 is mediated by activation of mTORC1, a negative regulator of TSC2, or inhibition of the mTORC1 subunit RAPTOR 5 [94]. A related study found that sterol response element binding protein 1 (SREBP1), a downstream effector of mTORC1, has a regulatory role in the development of ferroptosis [95]. SREBP1 acts as a transcription factor that regulates the transcription of several genes involved in lipid metabolism, including stearoyl-CoA desaturase (SCD1) [96]. SCD1catalyzes the formation of monounsaturated fatty acids (MUFA) from saturated fatty acids, and the production of MUFA can specifically inhibit the accumulation of lipid ROS on the plasma membrane, producing ferroptosis resistance [97]. Study confirms that inhibition of mTORC1 suppresses SREBP1 transcription and promotes ferroptosis sensitivity of cells [96]. In summary, inhibition of SREBP-SCD1 activity downstream of mTORC1 inhibits MUFA synthesis and thus induces ferroptosis. Taken together, MAPK inhibition can activate the LKB1-AMPK signaling pathway and induce ferroptosis by inhibiting mTORC1 activity.

In the process of regulating autophagy, there is a crucial protein of vesicle formation-BECN1 [98]. As the core component of class III phosphatidylinositol 3-kinase complex I (PtdIns3K) [99], BECN1 triggers different cell death by cooperating with different partners, and it is also a factor for occurrence of ferroptosis [100]. It was found that in the absence of glucose, AMPK was activated, and phosphorylated BECN1 at Thr388 to promoted the formation of the BECN1-PIK3C3 complex in autophagy, thereby inducing autophagy [100]. Meanwhile, activation of AMPK promotes phosphorylation of BECN1 at Ser90/93/96, which leads to the binding of BECN1 to the core component of system Xc^−^- SLC7A11 [101]. The formation of the BECN1-SLC7A11 complex blocks the activity of system Xc^−^, which leads to the inhibition of GSH synthesis and consequently the imbalance of amino acid metabolism, then inhibits the activity of GPX4 and thus induces ferroptosis [100]. In conclusion, inhibiting the MAPK pathway activates the LKB1-AMPK signaling pathway and thus promotes the binding of BECN1 to SLC7A11, inducing ferroptosis by blocking the activity of system Xc^−^.

### AMPK activates p53 to regulate ferroptosis

The tumor suppressor protein p53 (TP53), plays a critical role in the cellular response to stress, and different stress conditions that cause different states of p53 can lead to cancer cell survival or death. AMPK, an intracellular energy-sensing kinase, activates p53 through phosphorylation [102], while AMPK silencing attenuates the phosphorylation level of p53. In a study of the effect of aspartate (Asp)-asparagine (Asn) homeostasis on cell survival status, a positive feedback loop between p53 and asparagine metabolism was found. p53 inhibits ASNS expression by binding to the asparagine synthase (ASNS) gene, decreasing the Asp-Asn ratio, and direct binding of Asp to LKB1 activates AMPK while leading to higher activation of p53 [103]. Related studies have confirmed that LKB1 can be activated by AMPK and that sustained activation of AMPK can accelerate p53-dependent cellular senescence [102, 104]. While p53 has been classically recognized to impede cancer development by mediating cell cycle arrest, apoptosis, and senescence, new evidence suggests that p53 can induce ferroptosis in cancer cells by regulating metabolic activities. Traditionally, we are familiar with the idea that ferroptosis is mainly controlled by GPX4, but p53-mediated ferroptosis is GPX4-independent [105, 106]. As a new target for tumor therapy, the link between p53 and ferroptosis has attracted significant attention.

SLC7A11 is not only one of the critical factors in the development of ferroptosis but also one of the targets of p53 [52]. Related studies demonstrated that p53 inhibits system Xc^−^ activity by suppressing the expression of key protein SLC7A11, which leading to impaired cystine uptake and affecting the biosynthesis of GSH, and inhibiting GPX4 and ultimately leading to cell ferroptosis [52, 107]. The study has shown that p53, a novel regulator of histone H2B lysine 120 (H2Bub1), negatively regulates the level of (H2Bub1) by promoting the nuclear allosteric site of ubiquitin-specific processing protease 7 (USP7),H2Bub1 is enriched in the gene regulatory region of the SLC7A11 gene, the deletion of H2Bub1 significantly downregulates the mRNA and protein levels of SLC7A11, which increases the sensitivity of cells to ferroptosis [108]. Regulation of H2Bub1 by p53 also provides us with a novel mechanism for the induction of ferroptosis.

Maintenance of normal glutamate transport is also an important mode to avoid the occurrence of ferroptosis. Glutamine is converted to glutamate by glutaminases (GLS1 and GLS2), which drive ferroptosis through glutaminolysis [20]. As a critical enzyme in converting glutamine to glutamate, GLS2 (glutaminase 2) promotes the production of glutamate and α-Ketoglutaric acid by regulating glutamine metabolism, increasing mitochondrial oxidative phosphorylation and the generation of ATP [109]. High concentrations of glutamate inhibited system Xc^−^, a key transport system for ferroptosis, which make the synthesis of glutathione GSH inhibited, leading to a large accumulation of lipid peroxides and thus inducing the development of ferroptosis. Related studies reveal that the hepatocarcinogenesis was accompanied by reduced levels of GLS2 and found a reduced susceptibility to ferroptosis in GLS2 knockout cell lines, while the overexpression of GLS2 enhanced sensitivity to ferroptosis [110]. Decreased mRNA and protein levels of GLS2 were found in the WT cell line of SK Hep1, but not elevated in the p53KO cell line, giving us a hint of the relationship between p53 and GLS2 [110]. Related studies has confirmed that GSL2 is a target of p53-induced glutamine metabolism and that the GSL2 gene contains a binding element for functional p53 DNA in the promoter region, p53 binds to the GLS2 promoter and increases GLS2 expression under non-stress and stress conditions thereby enhancing mitochondrial respiration and ATP production [109, 111]. Thus, p53 enhances the expression of GLS2 and promotes the conversion of glutamate to α-ketoglutarate, which increases the production of lipid reactive oxygen species so that induce ferroptosis.

P53 can not only affect ferroptosis by directly acting on ferroptosis key transporter proteins and controlling GSH synthesis, but recently it was found that p53 can also induce ferroptosis by regulating polyamine metabolism [112]. The presence of polyamines is essential for the growth and survival of eukaryotic cells. As a rate-limiting enzyme in the catabolism of polyamines, spermidine/spermine N1-acetyltransferase 1(SAT1) is mainly responsible for catalyzing the acetylation of spermidine and spermine to form N^1^ -acetyl spermidine and N^1^-acetyl spermine, which is eventually converted back to putrescine or spermidine [112]. The process of polyamine catabolism is closely linked to the stress response to ROS, the reaction between SAT1 and polyamine oxidase (PAO) produces H_2_O_2_ and increases oxidative stress [113, 114]. Nutlin, a small molecule antagonist of MDM2, was found to activate p53 transcription, and mRNA expression of SAT1 was found to be significantly upregulated after using Nutlin on wild-type p53 melanoma cell line A375 [115]. Upregulation of SAT1 mRNA was also observed in p53 overexpressing cell lines [112]. Interestingly, in nutlin-acting SAT1 knockdown cell lines, the expression level of arachidonic acid lipoxygenase 15 (ALOX15) was radically found to be reduced, suggesting that ALOX15 may be a downstream effector of SAT1 acted by p53 [112]. It has been shown that ALOX15 acts as an "intermediate catalyst" to affect the occurrence of ferroptosis. Free PUFA is esterified to AA-PE, and ALOX15 can oxidize the esterified AA-PE to lipid peroxides, causing the accumulation of lipid peroxides and thus inducing the occurrence of ferroptosis [41]. Ferroptosis occurs in response to dual induction of SAT1 and ROS, and this process is accompanied by increased expression of ALOX15. In conclusion, the action of p53 activates the expression of SAT1 and increases the expression of ALOX15, which greatly promotes the oxidation of AA-PE and increases the production of lipid peroxides which causing ferroptosis [45, 112]. The activity of ALOX12, another member of the lipoxygenase family, has an influential role in the development of ferroptosis, and related studies have demonstrated that the chromosomal locus of the ALOX12 gene is very close to the p53 locus and that defects in the ALOX12 allele have been identified in human cancers [116]. U2OS cells treated with Nutlin and tert-butyl hydroperoxide (TBH), which can produce p53-dependent ferroptosis, but this ferroptosis was suppressed in ALOX12 knockout U2OS cells, suggesting that ALOX12 is required for p53-dependentferroptosis. ALOX12 specifically interacts with SLC7A11, to some extent also showing that p53 targeting of SLC7A11 induces ferroptosis dependent on ALOX12. Therefore, p53 can induce ferroptosis by inhibiting SLC711 and dependent on ALOX12 [105]. Calcium non-dependent phospholipase A2β (iPLA2β)-mediated lipid peroxide detoxification was found to inhibit ROS-induced ferroptosis independently of the GPX4 pathway, and inhibition of iPLA2β activity resulted in greater sensitivity of tumor cells to p53-promoted ferroptosis [106]. This also suggests that iPLA2β as a new target for the onset of p53-induced ferroptosis in cancer cells may provide a new entry point for cancer therapy. It is well known that p53 can induce the occurrence of ferroptosis, but ferroptosis negatively regulates p53. In the study of the mechanism related to the occurrence of p53-induced ferroptosis, it was found that p53 can inhibit the erastin-induced ferroptosis by inhibiting the localization and activity of dipeptidyl peptidase-4 (DPP4). Meanwhile, promoting the expression of the cyclin-dependent kinase inhibitor 1 (CDKN1A) which is mediated by p53 also plays an essential role in inhibiting the occurrence of ferroptosis [117].

**Fig. 2:**
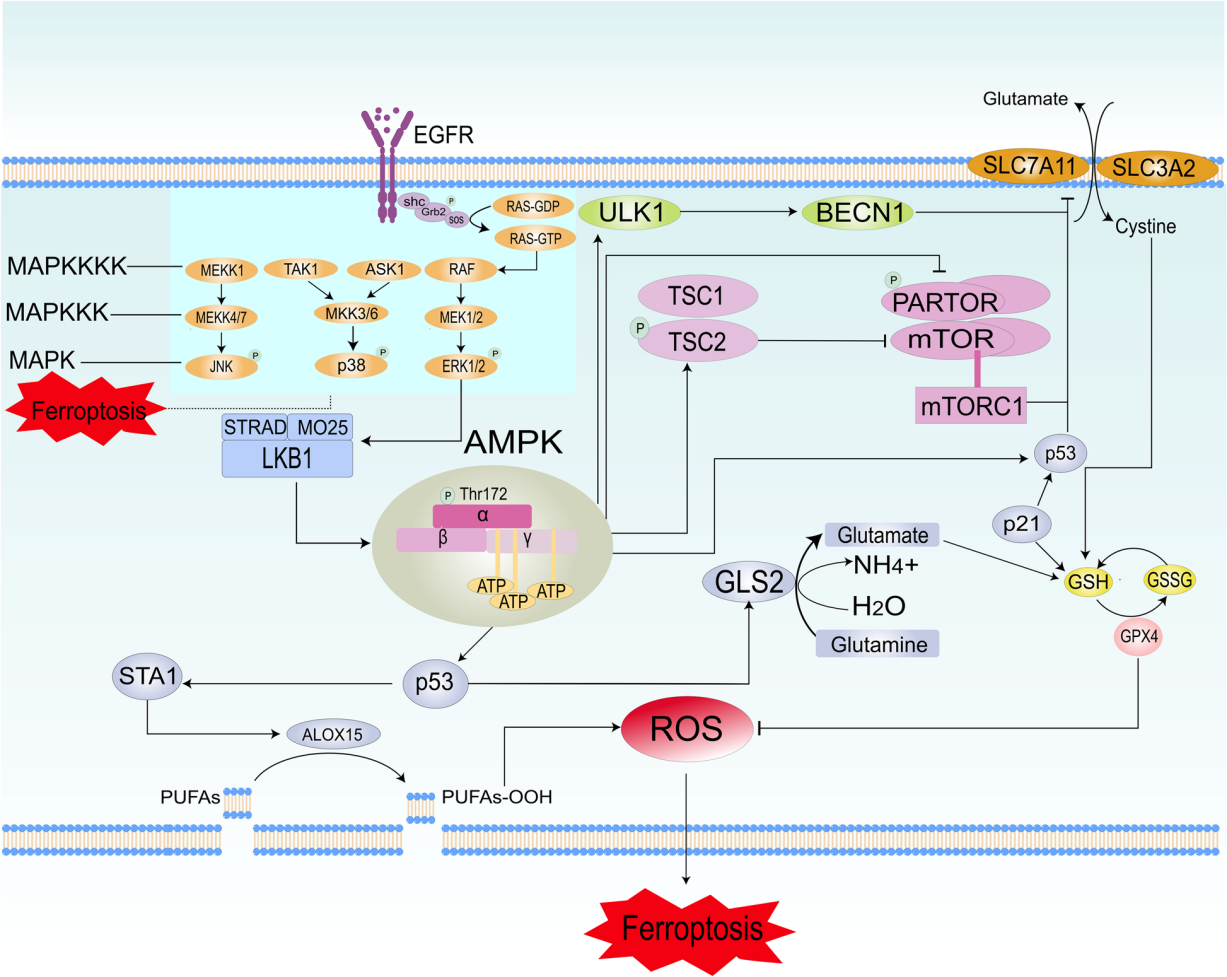
MAPK regulates the ferroptosis network

In conclusion, inhibition of the MAPK activates the activity of the LKB1-AMPK signaling pathway, which in turn activates p53 and induces ferroptosis. (Fig. 2).

## Ferroptosis activator

Numerous studies have confirmed that ferroptosis is crucial in killing tumor cells and inhibiting tumor growth, induction of ferroptosis has been widely recognized as a new cancer treatment strategy. Inhibiting the activity of the MAPK to active LKB1- AMPK can regulate the occurrence of ferroptosis through multiple signaling pathways, this ferroptosis regulatory network has great significance in cancer therapy, especially in drug-resistant tumors therapy. Here, we summarize some drugs that target this network to induce ferroptosis (Tables 1 and 2).

**Table 1:**
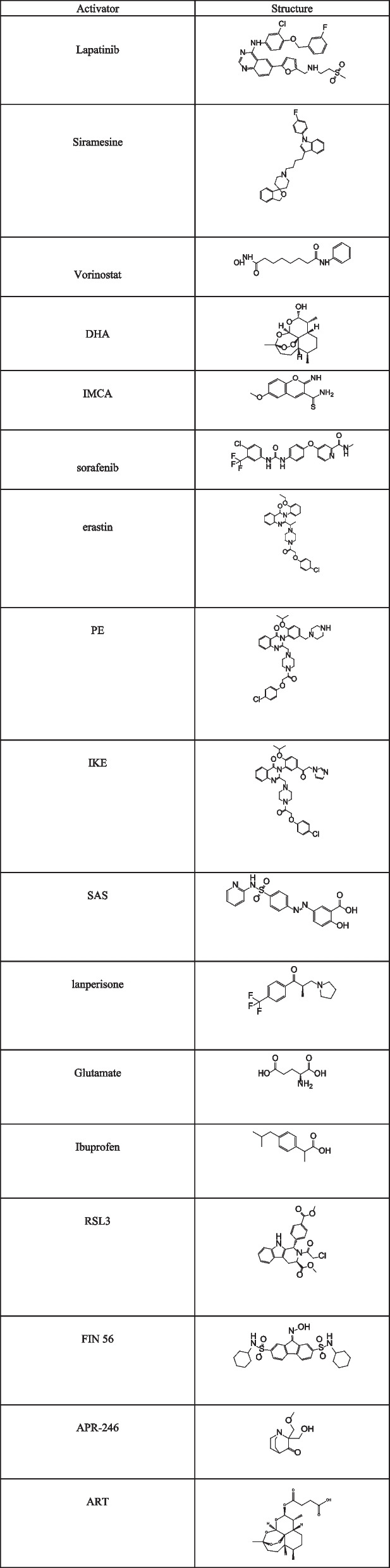
Chemical structure of some ferroptosis activators

**Table 2:** Action mechanism of ferroptosis activators

Classify	Inducers of ferroptosis	Mechanism of action	References
Targeting MAPK signaling pathway	Lapatinib + Siramesine	Blocking iron transport and increasing intracellular unstable iron, leading to increased ROS.	[118]
	Vorinostat	Inhibits system Xc^−^ activity, affects GSH synthesis, enhances the effect of ferroptosis inducers.	[119]
	Cetuximab + β-elemene	ROS accumulation, GSH depletion.	[120]
Targeting AMPK signaling pathway	DHA	Accelerate the degradation of ferritin, increase the content of unstable iron pool, promote the accumulation of ROS in AML cells and ultimately lead to ferroptosis.	[121, 122]
	IMCA	Inhibits system Xc^−^ activity, affects GSH synthesis, and leads to ROS accumulation.	[91]
Targeting system Xc-	sorafenib	Inhibits system Xc^−^ activity, affects GSH synthesis, and leads to ROS accumulation.	[17, 123]
	erastin	Inhibits system Xc^−^ activity, affects GSH synthesis, and leads to ROS accumulation.	[16]
	PE	Inhibits system Xc- activity, affects GSH synthesis, and leads to ROS accumulation.	[14]
	IKE	Inhibits system Xc^−^ activity, affects GSH synthesis, and leads to ROS accumulation.	[124]
	SAS	Inhibits system Xc^−^ activity, affects GSH synthesis, and leads to ROS accumulation.	[122, 125]
	LP	Inhibits system Xc^−^ activity, affects GSH synthesis, and leads to ROS accumulation.	[126]
	Glutamate	Inhibits system Xc^−^ activity, affects GSH synthesis, and leads to ROS accumulation.	[2]
	Ibuprofen	Inhibits system Xc^−^ activity, affects GSH synthesis, and leads to ROS accumulation.	[127]
Targeting GPX4	RSL3	Covalently binding GPX4 at the selenocysteine site to inactivates GPX4.	[10, 128]
	FIN56	Induces GPX4 degradation, binds to and activates SQS resulting in loss of CoQ10.	[14]
Targeting p53	APR-246	Induce ferroptosis in cells by disrupting the GSH/ROS balance.	[129]
	ART	Increases iron levels and accumulation of lipid peroxidation products, reduces GSH content.	[130, 131]

## Drugs induce ferroptosis by inhibiting the activity of the MAPK signaling pathway

As an upstream signaling molecule of MAPK, the EGF receptor (EGFR) activates the RAS-ERK1/2 signaling pathway [132]. EGFR activates the MAPK signaling pathway through two "arms", the C-terminal domain of EGFR binds to growth factor receptor binding protein-2 (Grb2) [133], the SH3 domain of Grb2 actively binds to the proline-rich carboxy-terminal tail phase of SOS, which is known that SOS is a RAS guanine nucleotide exchange factor, and EGFR binds to SOS by binding to Grb [134]. GTP-RAS recruits RAF kinases to the membrane and activates them, which activates the RAF-MEK-ERK-MAPK signaling pathway [135]. Furthermore, it was found that the activation of EGFR can enhance the expression of SLC7A11 in glioma cells, thereby enhancing the transport activity of system Xc^−^, resulting in ferroptosis resistance [136]. Thus, the selection of drugs targeting EGFR can inhibit the activation of the MAPK pathway and induce cell ferroptosis.

It was found that the anti-EGFR antibody cetuximab combined with β-Elemene can induce iron-dependent ROS accumulation, GSH depletion to induce ferroptosis and inhibit interepithelial transformation to kill KRAS-mutated metastatic colorectal cancer cells [123]. Lapatinib, a dual kinase inhibitor of EGFR and HER2 [137], which was used in combination with Siramesine to block the iron transport in treating breast cancer cells, which leads to an increase in ROS and the occurrence of ferroptosis [118]. The EGFR kinase inhibitor-Vorinostat can promote ferroptosis in EGFR-mutant lung adenocarcinoma cells by inhibiting SLC7A11 expression and enhancing the efficacy of ferroptosis activator [119].

### Activating AMPK signaling pathway to induce ferroptosis

A derivative of artemisinin, dihydroartemisinin (DHA), induces ferroptosis in acute myeloid leukemia (AML) cells. It was found that DHA induces autophagy by activating AMPK and regulating the activity of the mTOR/p70S6k signaling pathway. By accelerating the degradation of ferritin and increasing unstable iron pools, DHA promotes the accumulation of ROS in AML cells and ultimately leads to ferroptosis [121]. DHA can also induce lysosomal degradation of ferritin in an autophagy-independent manner, thereby increasing free iron in cells and making cells more sensitive to ferroptosis [138].

The benzopyran derivative 2-imino-6-methoxy-2H-chromene-3-carbothioamide (IMCA) inhibits the mTOR-p70S6K signaling pathway by activating AMPK. By down-regulating the expression of SLC7A11, inhibiting the activity of System Xc^−^, reducing the content of cysteine and glutathione, leading to the accumulation of reactive oxygen species and inducing ferroptosis in colorectal cancer cells [91].

### Targeting system Xc^−^ to induce ferroptosis

Sorafenib is a BRAF inhibitor that targets Raf-1 and B-Raf kinases, and sorafenib was found to induce ferroptosis [123]. However, sorafenib-induced ferroptosis was unrelated to its RAF kinase inhibition [139]. By inhibiting the activity of system Xc^−^, sorafenib blocks the transport of cystine/glutamate, leading to endoplasmic reticulum stress, impaired GSH synthesis, disruption of intracellular redox homeostasis and accumulation of lipid ROS, thereby inducing ferroptosis [17].

The first ferroptosis-specific activator, erastin, was found to induce ferroptosis by inhibiting the activity of System Xc^−^, which inhibit GSH synthesis [2, 16]. It was found that erastin can induce mitochondrial dysfunction by binding to the mitochondrial voltage-dependent anion channel (VDAC) and altering VDAC gating, resulting in abnormal iron ion metabolism and ultimately inducing ferroptosis [126]. The use of erastin in vivo is limited due to its poor water solubility and unstable metabolism, but its derivatives piperazine erastin (PE) and imidazole ketone erastin (IKE) both induce ferroptosis by inhibiting the activity of System Xc^−^ [14, 124].

Sulfasalazine (SAS) is synthesized from the antibiotic sulfasalazine and is commonly used to treat chronic inflammatory diseases [122]. It was found that SAS induces ferroptosis through inhibiting the uptake of cystine by System Xc^−^, leading to GSH depletion [125].

Lanperisone (LP) is a modified form of tolperisone that can effectively target Ras-mutated tumors. It was found that LP can induce ferroptosis by inhibiting the function of System Xc^−^ [126].

High glutamate concentrations induce ferroptosis in the brain and cancer cells by inhibiting System Xc^−^ [2].

Ibuprofen, a clinically used NSAID, induces ferroptosis in glioma cells through inhibiting System Xc- activity by down-regulating SLC7A11 expression [127].

### Targeting GPX4 to induce ferroptosis

RSL3 is a RAS lethal molecule that induces ferroptosis by inactivating GPX4 which covalently binding to selenocysteine at the active site of GPX4 [10, 128].

FIN56 induces post-translational degradation of GPX4 by relying on Acetyl CoA carboxylase, which decreases GPX4 expression and ultimately leads to cell ferroptosis. FIN56 can also activate squalene synthase (SQS), resulting in the deletion of coenzyme Q10 (coenzyme Q10, CoQ10), thereby enhancing the sensitivity of cells to ferroptosis [14].

### Targeting p53 to induce ferroptosis

Eprenetapopt (APR -246) is a p53 activator that restores wild-type p53 function in TP53- mutant cells. It was found that reactivation of the mutated p53 leaded to ferroptosis in the clinical treatment of acute myeloid whitehead disease and various solid malignancies [129].

**Fig. 3:**
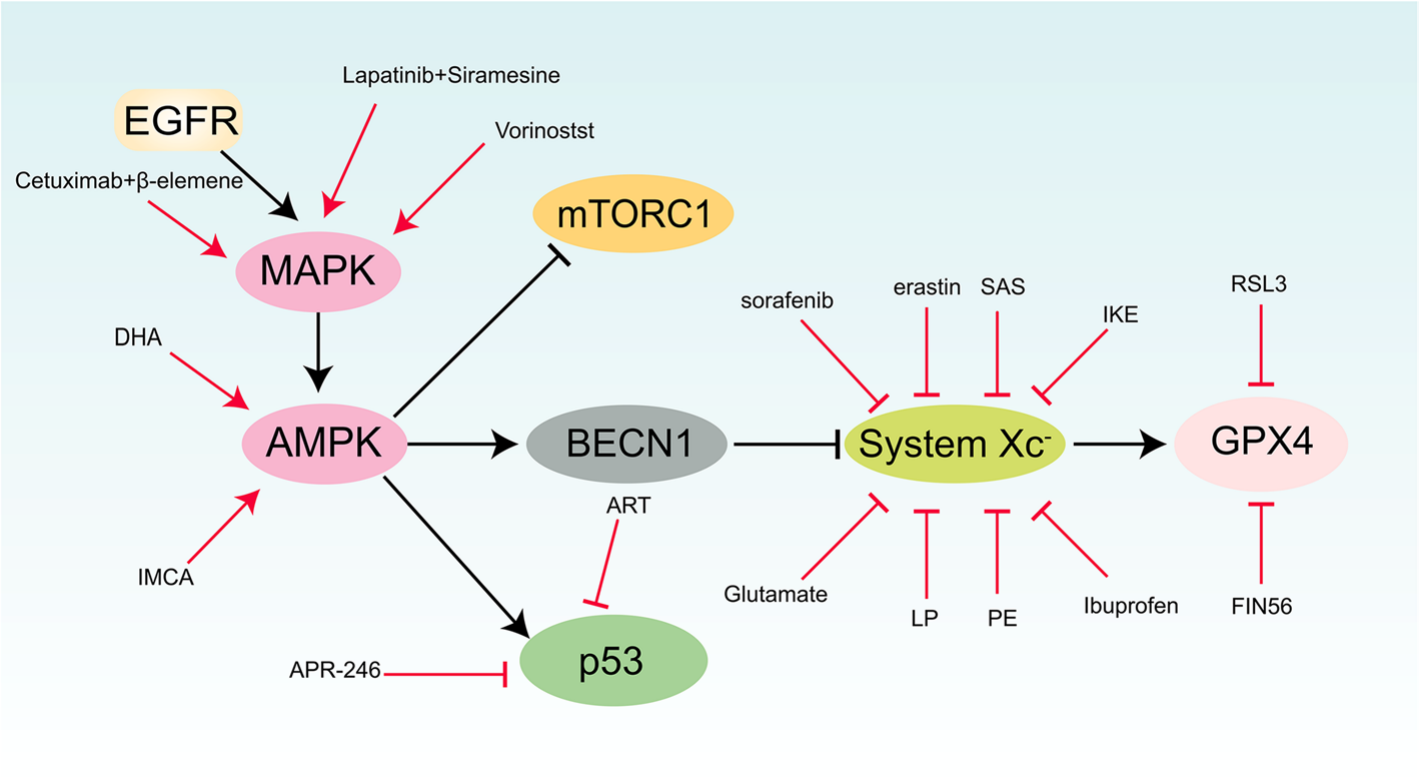
Target of drugs to induce ferroptosis in the MAPK-AMPK network

One of artemisinin's derivatives is artesunate(ART) can induce ferroptosis in pancreatic ductal adenocarcinoma cells which transformed by KRas, and the supply of exogenous iron will further enhance ferroptosis which induced by ART [131]. Another research has found that ART promoted the expression and nuclear import of p53 so that induces the occurrence of ferroptosis [131]. The study found ART can increase the level of iron and lipid peroxidation products, and reduce the GSH and NADPH content [130]. (Fig. 3).

## Conclusions and outlook

During the last decade or so, we have witnessed the discovery and development of ferroptosis and rapid progress in its mechanistic understanding. As a new potential modality of programmed cell death, it shows great potential and uniqueness in tumor therapy, and as a vast integrator of biological processes, this intricate web of relationships encompasses the occurrence of various pathophysiological processes. The number of relevant studies on ferroptosis has grown exponentially, with many cancer-related genes and signaling pathways exerting great potential in regulating ferroptosis, and mechanistic studies showing the potential evolutionary origin of ferroptosis.

There is growing evidence that ferroptosis is a complex network system, and the importance of MAPK as a key component of most signal transduction pathways and the most important common pathway for ferroptosis cannot be overstated. Here we enhance metabolic regulation with the help of LKB1 though linking MAPK to AMPK, followed by the effect of activating AMPK and thus p53 and mTORC1 signaling to influence the occurrence of autophagy-dependent ferroptosis and p53-activated ferroptosis. However, in a mechanistic study in the presence of cisplatin, we found that AMPK is not involved in LKB1-mediated p53 activation and that there is also a negative regulation of p53 activation through other mechanisms [140]. At the same time, but ignoring this complex process and focusing only on the direct relationship between LKB1-AMPK and ferroptosis, AMPK activation by the upstream kinase LKB1 can protect cells from lipid hydroperoxide accumulation and ferroptosis by inhibiting cellular lipid synthesis [141], which in turn gives a sense of contradiction in direction at the same time. We can see that in this complex network of signaling pathways, MAPK and AMPK are like double-edged swords controlling the direction of ferroptosis, and the summary of this complex network provides us with new ideas to study the mechanism of ferroptosis.

The existence of a complex network of MAPK-AMPK-ferroptosis provides a new vision for tumor therapy. The rapid development of ferroptosis-related research makes ferroptosis drugs promising as a promising cancer treatment, and the induction of ferroptosis can also reverse the phenomenon of drug resistance. We summarized various representative drugs for MAPK-AMPK-ferroptosis regulatory network, and also summarized targeted drugs for upstream of MAPK and downstream of AMPK. We considered that although inhibiting EGFR can inhibit the activity of MAPK pathway, the problem of acquired drug resistance of EGFR tyrosine kinase inhibitors (EGFR-TKI) has become a major clinical challenge, and overcoming the occurrence of drug resistance will also contribute to the induction of ferroptosis by MAPK-AMPK network. Ferroptosis-inducing drugs and other small-molecule compounds have the potential to become the pioneering force in tumor therapy. However, some of the drugs at this stage suffer from low bioavailability, insufficient targeting and poor stability, and the potential applications of these drugs need to be better understood. Further elucidation of the mechanism of action and characteristics of drugs targeting this regulatory network will have a profound impact on tumor therapy.

In conclusion, ferroptosis is like a compass in the desert that can show us the right path to the end of the road, and the complex mechanisms involved in deciphering these mechanisms are particularly important because of their uncertainty, and the discovery of these mechanisms will lead to appropriate and targeted therapeutic strategies. There is a long road ahead in the study of ferroptosis mechanisms, and these studies will eventually shed light on the unique significance of ferroptosis in nature.

## Data Availability

Not applicable.

## Abbreviations

MAPK: Mitogen-activated protein kinase
AMPK: AMP-activated protein kinase
ERK: Extracellular signal-regulated kinase
JNK: C-Jun N-terminal kinase
RCD: Regulated cell death
ROS: Lipid reactive oxygen species
DLBCL: Diffuse large B-cell lymphoma
RCC: Renal cell carcinoma cell lines
TfR1: Transferrin receptor 1
FTH1: Ferritin heavy chain 1
FTL: Ferritin light chain
NCOA4: Nuclear receptor coactivator 4
CdTe QDs: Cadmium telluride quantum dots
Fpn 1: Ferroportin 1
ASH: Subarachnoid hemorrhage
OSM: Oncostatin M
PE: Phosphatidylethanolamine
AA: Arachidonic acid
ACLS4: Acyl-CoA synthetase long-chain family member 4
LPCAT3: Lysophosphatidylcholine acyltransferase 3
ALOX15: Arachidonate lipoxygenase 15
ARF6: ADP-ribosylation factor 6
ROSI: Rosiglitazone
RKIP1: Raf1 kinase inhibitory protein
GSH: Free radical scavenger glutathione
system Xc^−^: Cystine/glutamate transpoter
SLC7A11: Disulfide-bonded heterodimer composed of a non-glycosylated light chain xCT
SLC3A2: A glycosylated heavy chain CD98hc
(18)F-FASu: Tracer (18)F-5-fluoro-aminosuberic acid
GSEA: Gene set enrichment analysis
Nrf2: Nuclear factor erythrocyte-related factor 2
NQO1: Quinone oxidoreductase 1
HO-1: Heme oxygenase-1
GSTA1: Glutathione S-transferase A1
γGCS: Glutamate-cysteine ligase
PEITC: β-Phenethyl Isothiocyanate
GNP: Gold nanoparticle
VDAC: Voltage-dependent anion channels
LKB1: Live kinase B1
CAMKK2: Ca (2 +)/calmodulin-dependent protein kinase kinase 2
LMP1: Latent membrane protein 1
ARNTL/BMAL: 1Aryl hydrocarbon receptor nuclear translocator-like
SIRT3: Sirtuin 3
IMCA: 2-Imino-6-methoxy-2H-chromene-3-carbothioamide
mTORC1: Mechanistic target of rapamycin complex 1
mLST8: Mammalian lethal with SEC13 protein 8
SREBP1: Sterol reaction element binding protein 1
SCD1: Stearoyl-CoA desaturase
MUFA: Monounsaturated fatty acids
PtdIns3K: Phosphatidylinositol 3-kinase complex I
TP53: Tumor suppressor protein p53
H2Bub1: Histone H2B lysine 120
USP7: Ubiquitin-specific processing protease 7
GLS2: Glutaminase 2
SAT1: Spermidine/spermine N1-acetyltransferase 1
iPLA2β: Independent phospholipase A2β
DPP4: Dipeptidyl peptidase-4
CDKN1A: Cyclin-dependent kinase inhibitor 1
EGFR: EGF receptor
Grb2: The growth factor receptor-bound protein-2
DHA: Dihydroartemisinin
